# Supplementary material for: Are carbon emissions trading and green financial instruments synergistic? -Comprehensive quantitative research based on content analysis
Source: PLoS One. 2024 Mar 7;19(3):e0298601. doi: 10.1371/journal.pone.0298601 (PMC10919601; doi:10.1371/journal.pone.0298601)
Supplement: S1 Data — (ZIP) [file pone.0298601.s001.zip › Data/地级市Z_F工作报告.zip!/╔╜╢1⁄2/╚╒╒╒╩╨/└┤╫╘╣1⁄2╓┌║┼í╛┬φ┐╦╩2╛▌═°í┐_2008.docx]

各位代表：

现在，我代表市人民政府，向大会作政府工作报告，请予审议，并请市政协各位委员和其他列席会议的同志提出意见。

一、过去五年政府工作回顾

本届政府履职以来的五年，是我市发展史上不平凡的五年。五年来，在省委、省政府和市委的正确领导下，在市人大、市政协的监督支持下，市政府团结依靠全市人民，坚持以邓小平理论和“三个代表”重要思想为指导，认真落实科学发展观，深入实施“四大战略”，积极构建和谐社会，圆满完成了市十五届人大一次会议确定的目标任务，有力地推进了日照科学发展、跨越发展、和谐发展。

五年来，我市经济快速攀升，综合实力显著增强。初步预计，全市生产总值由2002年的240亿元增加到2007年的600亿元，年均增长17.1%，比上一个五年提高5.6个百分点。人均GDP突破3000美元，是2002年的2.5倍。境内财政总收入由36.4亿元增加到149.69亿元，年均增长32.7%；地方财政收入2007年实现28.98亿元，是2002年的2.79倍。五年累计完成全社会固定资产投资1086.8亿元，是上一个五年的3.1倍。社会消费品零售总额由74.9亿元增加到170.5亿元，年均增长16.1%。金融机构存贷款余额分别由182.5亿元、165.8亿元增加到529亿元和539亿元，年均分别增长23%和27.3%。

五年来，我市结构调整成效明显，优势产业不断壮大。三次产业结构由22.5:36.4:41.1调整为13.3:51.2:35.5。农业综合生产能力逐年提高，茶叶、蔬菜、水产、畜牧等特色农业和农村二三产业快速发展，社会主义新农村建设扎实推进。工业化进程加快，钢铁、能源、机械制造、浆纸、食品加工、石油化工等临港工业迅速崛起，一批骨干企业培育壮大，成为经济增长的重要动力。全市规模以上工业企业由233家增加到813家，主营业务收入过亿元的企业由31家发展到118家；规模以上工业企业年主营业务收入、增加值、利税分别达到920亿元、235亿元、105亿元，年均分别增长45.7%、40.7%和58.2%。高新技术产业产值年均增长64.3%。物流、旅游、金融等服务业快速发展，日照港实现亿吨跨越，年货物吞吐量从3805万吨提高到13063万吨，跃升为我国大陆沿海第九个亿吨综合枢纽大港。

五年来，我市各项改革深入推进，对外开放迈出新步伐。农村税费改革顺利完成，各项综合配套改革稳妥推进。企业改革不断深化，现代企业制度逐步完善，各类要素市场快速发育。日照港、昌华集团分别在上海证交所、新加坡证交所成功上市。民营经济发展活力增强，日钢、五征、金马、三木、东升、晨曦等一批民营企业成为区域经济发展的骨干力量。财税、粮食、行政管理和事业单位等改革取得明显成效。全市五年累计实际利用外商直接投资6.87亿美元，是上一个五年的2倍。在我市投资发展的世界500强企业由3家增加到16家。进出口总值由9.23亿美元增加到55亿美元，年均增长42.9%，外贸依存度由29%提高到66%。成功承办了第六次泛黄海中日韩经济技术交流会议、中意企业合作高层论坛等重要活动，开通了至韩国平泽的客箱班轮航线，日照口岸落地签证获得国务院批准，与国外5个城市缔结友城关系，出入境外国人数从3.9万人增加到24.3万人。接轨青岛全面启动，与半岛城市群、鲁南经济带和沿亚欧大陆桥地区各市的合作取得新的成果。鲁南（日照）国际贸易与航运服务中心开工建设。青岛流亭机场在我市设立了虚拟空港，日照港与青岛港合资成立了集装箱码头有限公司。

五年来，我市基础设施建设加快，城乡面貌焕然一新。制定了城市发展战略规划，完成了新一轮城市总体规划修编，争取设立了岚山区，搭起了“双城一区、轴向拓展”的城市框架。五年累计完成市政建设投资107.5亿元，新增城市道路、绿化面积分别是上一个五年的4倍和5倍，森林覆盖率达到33.15%，人均公共绿地面积达到20.4平方米。建成了灯塔广场、世帆赛基地、水上运动基地、迎宾路、银河公园等城市精品工程，城市环境综合整治定量考核连续五年保持全省第一。港口规划建设取得重大进展，《日照港总体规划》通过交通部和省政府联合审查。五年累计完成港口建设投资118亿元，是上一个五年的5.4倍，先后建成矿石、集装箱等万吨级以上泊位20个，新增通过能力5530万吨。胶新铁路、同三高速、南北沿海公路建成通车，境内国道、省道全部升级改造；第一批百座水库除险加固工程全面完成，城市调水供水工程顺利实施，新增城市供水能力20万吨；新增变电容量126万千伏安，新建、扩建220千伏变电站5座。五年累计投入45亿元，大力推进农村“路水电医学”等设施建设，村村通硬化路率、自来水普及率、有线广播电视通村率分别达到98.5%、84%、87%，完成乡镇卫生院改造46所，新建改造农村中小学612处。

五年来，我市经济社会协调发展，人民生活水平显著提高。“科教兴市”战略深入实施，五年取得重要科技成果576项，国家专利申请量、授权量年均分别增长40%；争创中国名牌产品3个、中国驰名商标3个、山东名牌产品45个、山东著名商标24个，日照绿茶成为国家地理标志保护产品。小学、初中适龄儿童入学率均达到100%，高中段教育普及率比2002年提高38.9个百分点；大学科技园入园高校达到6所，在校大学生5.5万人。环境保护工作进一步加强，节能降耗、治污减排和节约集约用地取得初步成效。我市连续五年被省委、省政府授予人口和计划生育目标管理一等奖。卫生、文化、体育、广播电视等事业取得新的成绩。城镇居民年人均可支配收入由7423元增加到13000元，农民年人均纯收入由3121元增加到5240元，年均分别增长11.9%和10.9%；城乡居民储蓄存款余额达到250亿元，是2002年的2.2倍。五年累计新增城镇就业17.58万人，帮助4.2万名城镇失业人员实现再就业，有组织转移农村劳动力11万人。社会保障体系进一步健全，养老保险制度不断完善，城乡低保实现动态管理下的应保尽保，新型农村合作医疗参合率达到93.5%。实施经济适用房货币直补和廉租房补贴政策，五年拨付1.64亿元为3654户城市低收入家庭解决了住房困难。坚持不懈地为群众办实事，五年完成市办实事重点项目58件，有效解决了农民出行难、吃水难、看病难和困难群体生活难、就业难、子女上学难等切身利益问题。“平安日照”建设扎实推进，公众安全感进一步提高。

五年来，我市先后荣获中国优秀旅游城市、国家环保模范城市、国家卫生城市、国家园林城市、国家节水型城市、全国绿化模范城市、创建国家循环经济示范市和全国双拥模范城、中国金融生态城市、中国投资环境百佳城市、世界清洁能源奖等十几个先进称号，先后成功承办了国际欧洲级、470级帆船锦标赛、中国水上运动会和中国电影“金凤凰”奖颁奖系列活动等重大体育赛事和文化活动，城市竞争力、吸引力和影响力显著提升。我们欣喜地看到：日照的发展从未像今天这样充满活力，日照的城乡从未像今天这样美丽，日照的人民从未像今天这样充满自信，一个生机勃勃的新日照正在崛起！

在刚刚过去的2007年，市政府认真贯彻党的十七大和省、市党代会精神及宏观调控政策，紧紧抓住省委、省政府构建“一体两翼”发展格局、加快鲁南经济带规划建设等重大战略机遇，乘势而上，奋力拼搏，经济社会实现了又好又快发展。与上年相比，全市生产总值增长17%，地方财政收入增长39.6%，规模以上工业增加值、实现利税、利润分别增长30%、98%和80.7%，规模以上固定资产投资增长25%，社会消费品零售总额增长17.5%，进出口总值增长40.5%，城镇居民人均可支配收入和农民人均纯收入分别增长18%和13%，主要经济指标增幅均居全省前列。同时，万元GDP能耗、万元工业增加值取水量和化学需氧量、二氧化硫排放等节能降耗减排指标均呈下降趋势。主要做了以下八个方面的工作：

（一）大力发展临港工业、现代服务业和高新技术产业。紧紧抓住结构调整这条主线，以招商引资为突破口，全力推进临港工业大项目建设。先后组织赴境内外重点地区开展投资环境推介和招商引资活动，收到良好效果。全年共引进投资过亿元的项目107个，实际外商直接投资2.3亿美元，增长69.1%。总投资4.9亿美元的现代威亚汽车发动机项目一期工程建成投产，投资14亿美元的亚太森博浆纸二期项目、投资44亿元的华能日照电厂二期工程和中储粮油、华海造船、锦湖化工等一批重点项目相继开工建设。38家临港工业企业实现主营业务收入、利税、利润分别占全市规模以上工业总量的65.6%、80.9%和81.3%。大力发展服务业，积极培育港口、旅游、体育等特色经济。举办了日照港口服务内地经济系列推介会，加强港企、港港合作，促进了港口业务快速增长。全年完成集装箱运量43万标箱，比上年增长70.2%。通过举办中国水上运动会、黄河经济协作区旅游经济合作峰会等活动，加强旅游与文化体育结合，旅游人数突破1200万人次，旅游收入突破65亿元。开工建设了安东卫海货水产城、龙鼎花生电子商务、新加坡衡悦海曲等商用设施和项目，营业额过亿元的商业零售企业达到7家。大力发展高新技术产业，高新技术产业产值增长65%，占规模以上工业总产值的比重上升3.2个百分点。

（二）加快推进县域经济和新农村建设。县域经济发展速度、质量、后劲同步提升，全年县域生产总值、规模以上固定资产投资、地方财政收入分别比上年增长24.2%、28.9%和37.8%。围绕增加农民收入，大力发展现代农业，不断提高产业化、标准化、品牌化水平。全市粮食总产达到104万吨，高效经济作物面积发展到200万亩，水产品总产量达到63.2万吨，畜牧业产值占农业总产值比重提高到22%。市级以上农业龙头企业发展到108家，美佳集团成为国家重点农业产业化龙头企业，无公害农产品和绿色食品认证达到98个。编制完成了新农村建设总体规划，示范试点工作扎实推进。加大农村基础设施和生态环境建设投入，农民生产生活条件进一步改善。认真落实各项支农惠农政策，全年发放粮食直补资金1843万元，生产资料综合直补4015万元，库区移民后期扶持资金11200万元，渔用等燃油补贴9283万元，良种、农机具购置等补贴610万元。

（三）加强节能减排、环境保护、集约节约用地工作。以钢铁、水泥、电力、焦炭、造纸5大行业和28家耗能企业为重点，深入开展“双高”行业专项清理，对违规企业实行停产限期治理。以严查环境违法、保障群众健康为主题，扎实开展环保集中整治活动，对粉尘污染、废气排放、脱硫设施建设滞后等问题进行重点整治，对未经审批、无治污设施、污染严重和饮用水源地周围不能达标排放的企业实施了关停。发布实施了《日照市循环经济促进办法》，有1个园区、13家企业被列入省重点推进的循环经济园区和循环经济企业。市政府先后发布三个通告，对海岸带、市区饮用水源地和卧龙山区域实行严格的控制和保护。集中开展土地执法“百日行动”，依法查处违法占地行为。加强基本农田保护，耕地总量保持了动态平衡。大力发展节地型经济，通过盘活闲置土地、滩涂开发等增加用地指标6000余亩，有效缓解了用地紧张的压力。

（四）抓好城乡环境和基础设施建设。编制完成了市区北部、滨海岸线、涛雒等重点地区规划，市区详规覆盖率达到100%。投资31亿元，组织实施30项城市建设重点项目，完成了日照奥林匹克水上公园、城市污水再生利用、沙墩河环境整治、临沂路绿化等重点工程。出台了关于城中村改造的试行意见，启动了两个村的试点工作。加强城市执法管理，深入开展城乡环境综合整治，完成了3个旧居民小区、10条背街小巷、8条次干道和10条污水沟的整治改造，建成户用沼气池3840个，人民群众生活环境得到改善。坚持适度超前，加大港口、电力、公路和园区的建设力度。投资26.1亿元，新建续建了西港区二期、矿石码头二期、中心渔港、岚北港区原油码头等13项港口重点工程。开工建设了北水南调二期、沭河综合治理等工程，完成了335省道市区至陈疃段的改造升级。经过全市人民的共同努力，顺利通过了中国人居环境奖的考核验收。

（五）大力加强财税金融工作。深入推进财源建设，大力培植骨干财源和新兴财源。加强税收和非税收入征管，完善综合治税和诚信纳税体系，加大土地出让金征缴和清欠力度，财税收入大幅度增长，结构明显优化。全年地方财政收入比上年净增8.22亿元，税收收入占地方财政收入的比重达到83%，比上年提高2.2个百分点。积极推进财政改革，加强公共财政建设，不断优化支出结构，全年各级财政用于民生的投入达到11.7亿元。金融运行状况良好，对地方经济的支持力度不断增强。金融机构各项存贷款余额分别比年初增加109亿元和89亿元，异地银行贷款余额达到72亿元。市商业银行在全国110家城市商业银行竞争力排名第三位。保险业服务领域不断拓宽，全年实现保费收入12亿元，比上年增长27.9%。

（六）认真解决民生问题。就业和社会保障工作进一步加强，全年新增城镇就业3.97万人，城镇登记失业率稳定控制在3.5%以内。积极推进新型农村社会养老保险试点和被征地农民、农民工、城镇灵活就业人员的基本养老、医疗保险工作。完善城乡社会救助体系，提高了城乡低保对象补助标准，全年发放保障金2500万元。承诺的12件为民实事顺利完成：全部免除30.2万名城乡义务教育阶段学生的杂费，对2.9万名贫困家庭学生免费提供教科书，为3721名贫困家庭学生补助寄宿生生活费；投资8500万元，建成531个村的自来水工程，新增受益人口36.5万人；投资1851万元，对13处重点乡镇卫生院进行整修改造，建成44处社区卫生服务场所；投资709.5万元，实施了助明、助听、助行、助医、助居“五助惠残”工程，受益残疾人2273人；投资1.58亿元，建成乡镇客运站19处，新建、改建农村公路320公里；投资2000余万元，对25处乡镇敬老院进行了改扩建，“五保”对象集中供养率达到60%；积极推进“万村千乡市场工程”，加强农村“一个网络、两个平台”建设，新建农村社区服务中心12处、超市45处，改造规范农家店、农资专卖店312个。深入开展农产品质量和食品、药品安全专项整治，人民群众饮食、用药更加安全。重视做好信访工作，坚决纠正损害群众利益的行为；坚持重心下移、一线布警、打防结合，确保了社会治安局势持续平稳；认真抓好安全生产，严防各类重大事故发生，保障了人民群众生命财产安全。全市刑事案件发案、安全生产事故起数分别比上年下降10%和20%，全年没有发生重大安全生产事故。

（七）努力推动社会事业协调发展。基础教育、职业教育、成人教育和高等教育统筹发展，办学质量不断提高。市实验学校、日照港中学迁建工程相继完成，补充农村中小学校教师471人，安置外来务工人员子女入学3016人。承办了第四届中国新闻摄影“金镜头”颁奖等文化活动，启动了莒县博物馆新馆建设，建成了市档案馆，文化遗产保护工作得到加强。成功承办了2007年中国水上运动会，“安全水运、创新水运、奥运水运、生态水运、开放水运、欢乐水运”的特色得到各方好评。人口自然增长率控制在3.8‰以下，圆满完成了省委、省政府下达的控制指标。全民普法与依法治市深入推进，法律服务、法律援助水平明显提高。基层民主政治建设得到加强，“村务大事村民公决”荣获全国村务公开民主管理制度创新奖，新建城市社区8处。广泛开展以社会主义荣辱观教育为主题的文明创建活动，涌现出“滴水工程”慈善品牌和救人英雄孙靖杰等先进典型。重视做好国防动员、民兵预备役、优抚安置和双拥共建工作，外事、对台、气象、物价、史志、人防等在服务经济社会发展中发挥了积极作用，妇女儿童、新闻出版、广播电视、民族宗教、老年人、红十字等工作取得新的成绩。

（八）着力提高政府管理服务水平。坚持依法行政，自觉接受人大、政协及社会各界监督。全年共办理人大代表建议46件、政协委员提案190件，办复率、满意和基本满意率均达到100%。围绕加强社会管理和公共服务，先后制定出台了关于做好社会保障、节能减排、农民教育培训、农民工、社区建设、应急管理和加强农村最低生活保障、安全生产工作等一系列文件。组织制定了128项市级公共安全应急预案，启动了市级应急指挥平台一期工程建设，开展了12次大型应急处置综合演练活动，应急管理体系建设走在全省前列。积极推行充分授权、市区联合审批等制度，精简行政审批事项85项。全面落实政府信息公开、新闻发布会、重大社会公共事项决策听证等制度，政务公开和科学民主决策机制不断完善。坚持市长接听日制度，做好市长公开电话、市长信箱、“行风在线”直播等方面的工作，民意渠道进一步畅通，解决了一批事关群众切身利益和社会关注的问题。按照市委的部署要求，深入开展“作风建设年”活动，政府工作人员公仆意识、服务意识、法制意识、责任意识进一步增强，行政服务效能有了新的提高。落实党风廉政建设责任制，强化行政监察和审计监督，深入开展商业贿赂专项治理，从严查处违法违纪案件，全年共处分违反政纪人员18人，初步形成了廉洁勤政的政务环境。

在肯定成绩的同时，我们也清醒地看到，当前日照在加快发展中还面临不少困难和矛盾：经济总量仍然较小，人均占有还低于全省平均水平，结构性矛盾比较突出，高新技术产业、现代服务业发展不够快；经济发展方式比较粗放，自主创新能力还不强，资源利用率较低，节能减排降耗压力增大；对外开放水平与沿海开放城市的地位还不相适应，特别是利用外资规模较小，出口商品结构有待优化；县域经济仍较薄弱，县乡财政还比较困难，农民持续增收的基础尚不稳固；消费价格指数上涨压力增大，部分群众在生活、就业、就医、教育、社保、住房等方面还存在困难；政府机关自身建设需进一步加强，个别部门和少数工作人员服务意识弱、工作效率低。我们一定高度重视存在的问题，采取有力措施，认真加以解决。

各位代表，过去五年，我们历经考验，克服困难，拼搏进取，进一步开创了日照改革开放和现代化建设的新局面。回顾五年的奋斗历程，我们深刻体会到，要把日照的改革、发展和各项事业不断推向前进，必须坚持以科学发展观统领全局，牢牢抓住发展这个第一要务，努力追求又好又快发展；必须坚持解放思想，创新发展理念，积极探索具有日照特色的发展路子；必须坚持改革开放，为各项事业注入新的生机与活力；必须坚持以人为本，把群众利益放在首位，做到发展为了人民、发展依靠人民、发展成果由人民共享；必须坚持真抓实干、埋头苦干，努力创造对得起人民、无愧于历史、经得起检验的业绩！我们深知，五年来取得的成就，得益于省委、省政府的亲切关怀，得益于市委的正确领导，得益于人大、政协和社会各界的监督支持，得益于历届市领导班子打下的良好基础，是全市人民团结奋斗的结果。在此，我代表市人民政府，向辛勤工作在各条战线的全市人民，向各级人大代表、政协委员，向各民主党派、工商联、无党派人士、各人民团体，向离退休老领导、老同志，向驻日照人民解放军和武警官兵，向所有关心支持日照建设的社会各界人士和海内外朋友，表示崇高的敬意和衷心的感谢！

二、今后五年的奋斗目标和主要任务

未来五年是我市全面建设小康社会、为提前基本实现现代化奠定坚实基础的重要时期，也是在新的起点上推进又好又快发展、实现富民强市新跨越的关键时期。在前进的道路上，我们面临的机遇前所未有，挑战前所未有，机遇大于挑战。经济全球化、区域经济一体化深入推进，生产要素跨国流动不断加快，日韩产业向山东半岛加速转移，为我市扩大开放特别是打造新兴的外资聚集区带来了难得的机遇。党的十七大对推进中国特色社会主义伟大事业作出全面部署，为我们继续解放思想、坚持改革开放、推动科学发展、促进社会和谐指明了前进方向。省委、省政府作出了构建“一体两翼”发展格局、加快鲁南经济带规划建设和建设海洋经济强省的重大决策，要求我市发挥港口带动作用、当好鲁南对外开放的窗口，为我们加快发展提供了战略机遇。特别是经过建市18年的不懈努力，日照加快发展的物质基础更加坚实、设施条件日臻完善、内生力量不断增强，我们有机遇、有条件、有能力发展得更好更快。

今后五年政府工作的总体要求是：全面贯彻落实党的十七大、省九次党代会和市十一次党代会精神，高举中国特色社会主义伟大旗帜，以邓小平理论和“三个代表”重要思想为指导，以科学发展观统领全局，坚持好字优先、好中求快，在新的起点上大力实施“港口立市、工业强市、科教兴市、生态建市”战略，更加注重改革开放，更加注重转变发展方式，更加注重节能减排，更加注重城乡统筹，更加注重改善民生，加快工业化、信息化、城镇化、市场化、国际化进程，全力推动科学发展、跨越发展、和谐发展、又好又快发展，努力构建“富裕日照、文明日照、平安日照、生态日照”，为早日跨入全省先进行列而努力奋斗。主要目标任务是：

——人均生产总值提前翻两番。坚持速度质量效益相协调，消费投资出口相协调，人口资源环境相协调，发展改革稳定相协调。在优化结构、提高效益、降低消耗、保护环境的基础上，主要经济指标增幅继续保持全省领先水平，综合经济实力位次前移。到2012年全市生产总值达到1300亿元，年均增长15%；到2011年人均生产总值达到36600元，提前实现人均国内生产总值比2000年翻两番的目标。

——优势产业和特色经济更加突出。坚持以先进制造业、高新技术产业、现代服务业为重点，加快产业结构优化升级，努力打造山东半岛、鲁南经济带新兴的先进制造业基地和临海产业区。以临港工业、临港服务业为重点的港口经济更为活跃，支撑拉动作用更加明显；海洋经济、旅游经济等特色经济发展加快；“水上运动之都”的城市品牌进一步提升。到2012年三次产业结构调整到7：54：39；临港产业增加值占生产总值的比重达到50%以上；日照港货物吞吐量达到2.4亿吨，集装箱运量突破220万标箱。

——经济发展方式明显转变。努力建设资源节约型、环境友好型社会和创新型城市，加强生态文明建设，基本形成与节约能源资源和保护生态环境相适应的产业结构、增长方式、消费模式。高新技术产业产值占规模以上工业总产值的比重力争达到30%；万元GDP能耗降低23%，化学需氧量、二氧化硫排放总量分别削减9%、18%，工业用水重复利用率达到90%；城市生活垃圾无害化处理率达到100%，城市生活污水处理率提高到85%，中水回用率达到35%，森林覆盖率达到36%。

——经济市场化、国际化程度大幅提高。坚持市场取向改革，更好地发挥市场在资源配置中的基础性作用，服务鲁南经济带和新亚欧大陆桥的区域要素市场初步形成。重要领域和关键环节改革迈出重大步伐，充满活力、富有效率、创新开放、有利于科学发展的体制机制基本形成。坚定不移地扩大对外开放，全面提升招商引资水平，加快转变外贸增长方式，使外向型经济达到新水平。争取到2012年全市实际利用外资达到5亿美元，年均增长20%，进出口总值年均增长20%。

——城乡发展更加协调。更加注重统筹城乡发展，全面推进城乡建设，逐步完善基础设施和公共服务体系，城镇化水平达到50%以上。县域经济实力明显增强，各区县在全省综合排名的位次大幅度前移，有1—2个区县跨入全省30强。农村各项改革全面深化，农村公共事业建设得到加强，社会主义新农村建设步伐加快，初步形成工业反哺农业、城市支持农村的城乡一体化格局。

——民生状况得到更大改善。坚持以人为本，富民优先，努力解决好人民群众最关心、最直接、最现实的利益问题。城乡居民受教育程度普遍提高，劳动就业比较充分，社会保障体系更加完善，生态环境明显改善，民主法治日益健全，人民生活更加宽裕，家庭财富普遍增加，人人享有基本公共服务，社会成员的幸福感进一步提高。到2012年，城镇居民人均可支配收入和农民人均纯收入分别达到18500元和8400元，年均增长8%以上。城镇登记失业率控制在3.5%以内，城镇基本社会保障覆盖率达到97%以上。

各位代表，盼发展、求和谐、强实力、富民生，是282万日照人民的共同愿望。我们坚信，只要全市人民解放思想，抓住机遇，同心同德，真抓实干，就一定能够战胜前进道路上的一切困难，在新起点上实现富民强市的新跨越！

三、2008年政府工作任务

实现今后五年的目标任务，首先要扎扎实实地做好2008年的工作，确保开好局、起好步。我们将以全面贯彻党的十七大、中央经济工作会议和省委九届三次全会精神为主线，以科学发展观统领经济社会发展全局，坚持好字优先、好中求快，按照防过热、防通胀的宏观调控要求，着力推进改革开放、优化经济结构、加强节能减排、优先改善民生、促进社会和谐，努力保持经济社会又好又快发展的良好势头。今年全市经济和社会发展的主要预期目标是：生产总值增长15%，地方财政收入增长17%，规模以上固定资产投资增长20%，社会消费品零售总额增长16%，规模以上工业增加值增长18%，进出口总值增长20%，城镇居民人均可支配收入增长9%，农民人均纯收入增长8%。主要约束目标为：万元GDP能耗降低5%，二氧化硫排放减少4.68%，化学需氧量排放降低3.66%，人口自然增长率控制在6‰以内。重点抓好以下五个方面的工作：

（一）统筹城乡发展，扎实推进新农村建设。我市农村人口占总人口的80%以上，农业的基础地位尤为重要，任何时候都必须把“三农”工作放在重中之重的位置。要坚持以增加农民收入为中心，以发展现代农业、繁荣农村经济、改善农村环境为重点，因地制宜，量力而行，扎实推进新农村建设。

一是加快发展现代农业。实行最严格的耕地保护政策，切实稳定粮食、油料和肉蛋奶等主要农产品生产。围绕构建现代农业产业体系，大力推进集约化、产业化、标准化、品牌化生产，加快建设一批特色农产品基地和出口农产品基地。加大对重点农业龙头企业扶持力度，积极发展各类农民专业合作组织。加强动植物疫病防控和水产品质量检测，加快农产品质量监测中心建设，提高农产品质量安全水平。完善农业科技推广体系，加大农作物良种补贴力度，加快发展农业机械化，鼓励发展民营经济、农产品加工企业和流通服务业，广开农民增收渠道。以培养有文化、懂技术、会经营的新型农民为重点，大力实施农民教育培训工程。年内完成各类培训6.5万人次，有组织转移农村劳动力2万名。

二是加强农村基础设施和社会事业建设。巩固农村“路水电气医学”建设成果，加强农民生产服务体系、生活服务体系、社会保障服务体系建设。年内投资1200万元，改造建设标准化村卫生室800所；各级财政投资1.61亿元，将新型农村合作医疗人均补助额提高到80元；投资2400万元，新建改造120个村的自来水工程，全市农村自来水普及率提高到88%；完成农村公路改造200公里，重点解决偏远村庄群众行路难的问题；开工建设小仕阳、长城岭两座大中型水库和90座小型水库除险加固工程，继续搞好农村改水、改厕、改灶、改圈；加大“五保”对象集中供养力度，年内集中供养率提高到70%以上；扎实推进农村现代流通服务体系建设，新建农村社区服务中心10处、综合超市35处、农家店230个。充分发挥小城镇在推进城乡统筹中的节点作用，加快建设一批规划科学、规模适度、经济繁荣、管理有序的中心镇、特色镇，引导和鼓励农民向小城镇集聚。

三是深化农村综合改革。全面落实多予少取放活的方针，建立健全防止农民负担反弹的长效机制，切实维护农民权益。稳定完善农村土地承包关系，规范土地承包经营权流转，鼓励有条件的地方发展适度规模经营。深入推进乡镇机构、县乡财政、农村金融管理体制改革，完善“以县为主”的农村义务教育管理体制，积极化解农村义务教育的历史债务。按照“三个明显高于”的要求，加大“三农”投入，落实好粮食直补、生产资料综合补贴、大中型水库移民后期扶持等各项强农惠农政策。各级财政今年安排用于“三农”的资金17.68亿元，比上年增长14%。

四是加快提升县域经济综合实力。以壮大县乡财力、增加农民收入为目标，以工业为主导、开放为动力、农业为基础、城镇和园区为支撑，加快发展县域经济。充分发挥港口、资源、产业等优势，大力招商引资，积极引进临港工业配套型、产业协作型、农产品加工带动型、财税增收型项目，加快培育特色支柱产业和支撑拉动作用强的骨干企业。大力发展镇村经济，鼓励具备条件的乡镇率先发展。加大对县域经济的政策扶持力度，统筹市县基础设施建设，促进县域经济更好更快发展。全年县域规模以上工业增加值、固定资产投资分别增长20%和25%以上。

（二）着力转变经济发展方式，推动经济又好又快发展。坚持以现代产业体系和新型工业化为主导，认真组织实施市政府《关于推进全市产业结构优化升级的意见》，促进经济增长由主要依靠投资拉动向依靠投资、消费、出口协调拉动转变，由主要依靠第二产业带动向三次产业协调带动转变，由主要依靠增加物质资源消耗向主要依靠科技进步、劳动者素质提高和管理创新转变。

一是大力发展临港先进制造业。主动对接省委、省政府构建“一体两翼”发展格局、加快鲁南经济带开发建设的战略部署，积极争取在我市规划建设鲁南临海产业区，着力培育优势产业、核心企业和特色经济。按照“大项目—产业链—产业基地”的发展方向，抓好现代威亚发动机二期、亚太森博浆纸二期、华能日照电厂二期等大项目建设，做好省政府规划的日照大型钢铁精品基地建设筹备工作，加快培育冶金、石油化工、能源、浆纸、机械制造、食品加工和修造船等临港产业。依托钢铁、发动机、造船等骨干项目，培育关联配套产业，促进企业集聚和产业集群发展。

二是加快发展服务业。坚持以市场化、产业化、品牌化、国际化为方向，拓展生产性服务业，丰富消费性服务业，提升农村服务业，壮大现代服务业，重点发展港口物流、商贸服务、旅游会展、文化体育、房地产、科技信息、金融保险、商务服务“八大产业”，集中力量培育10个重点园区、20家重点企业和10个重点市场“三大载体”，努力把服务业打造成为国民经济的主导产业、就业增加的主要载体、财政增收的重要来源。港口全年货物吞吐量确保达到1.5亿吨，力争达到1.6亿吨；集装箱运量确保达到80万标箱，力争突破100万标箱。旅游人数、旅游收入分别增长18%和25%。把扩大消费需求作为促进服务业发展的重要措施，进一步落实消费政策，拓宽消费领域，开拓农村市场，积极引导住房、汽车合理消费，扩大通信、旅游、休闲、文化、健身等热点消费，努力以消费促进经济增长。

三是积极推进自主创新和高新技术产业。抓住科技成果转化、科技公共服务平台建设、科技人才队伍培养等关键环节，加快创新型城市建设。全面落实促进企业自主创新的政策措施，完善以企业为主体、市场为导向、产学研相结合的技术创新体系。年内新增10个市级工程技术研究中心，专利申请量、授权量均增长20%以上。加强知识产权的开发、应用和保护，大力引进高层次人才和紧缺人才。注重发挥大学科技园和日照高新技术产业园区的创新服务功能，吸引高科技企业集团和研发机构入园，加快科技成果孵化基地建设，深化产学研合作，努力打造我市科技创新的平台。合理开发和保护海洋资源，加快发展海洋科技、海洋经济，努力建设海洋经济强市。大力发展高新技术产业，重点培植电子信息、生物工程、精细化工、新材料、新能源及环保产业，开发引进软件、光电子、生物医药、先进装备等项目。全年高新技术产业产值达到180亿元，占规模以上工业产值的比重提高3个百分点以上。

四是加强节能减排和节约集约用地工作。认真落实国务院《节能减排统计监测及考核实施方案和办法》，强化统计、监测和考核“三个体系”建设，打好节能减排攻坚战，切实把资源利用率提上去，把能耗和污染排放降下来。抓好冶金、建材、电力、化工、造纸等重点行业和50户重点企业的节能降耗，加大重点流域、重点区域污染治理和落后产能淘汰力度，加快推进污水处理厂、燃煤电厂脱硫等重点治污工程建设。认真实施循环经济发展规划，大力发展循环型工业、循环型生态园区，推广使用新能源、清洁能源和可再生能源，提高企业清洁生产水平。严格执行建筑节能标准，推进社会节能。发展节水型产业，严格限制地下水开采，推广中水回用、海水淡化和直接利用。加强土地管理，鼓励建设节地型项目，依法加大对闲置土地的处置力度，提高土地节约集约利用水平。

（三）大力推进改革开放，不断增强发展动力。改革开放始终是发展的活力源泉。必须坚持创新驱动，用改革的办法解决前进中的矛盾和问题；必须发挥开放优势，加大招商引资力度，全面提高对外开放水平，努力为经济发展注入活力、拓展空间、增强后劲。

一是深入推进各项改革。深化企业改革，建立完善现代企业制度，重视解决已改制企业特别是破产企业的遗留问题。大力发展民营经济，鼓励民间资本进入基础设施等领域。推进事业单位分类改革，完善事业单位社会保险和用人制度。深入推进财税体制改革，全面落实新的企业所得税法，深化部门预算、国库集中支付、政府采购和收支两条线改革。强化财政监督检查，健全资金管理制度和绩效考评办法，加强国有资产及收益管理。加强诚信体系建设，完善创新财源建设和综合治税体系。加快金融、资本市场建设，培育壮大地方金融机构，吸引国内外各类银行进入我市。健全征信体系，营造良好的信用环境，防范和化解金融风险。深化投资体制改革，开发和引入产业投资基金、建设债券等融资产品，推动重点产业和重点项目建设。积极培育上市资源，加快企业上市步伐，扩大直接融资规模。

二是切实提高招商引资的质量和水平。坚持“引进来”和“走出去”相结合，年内组织赴韩、日、欧美、新加坡、港台等地区和境内江浙沪粤重点城市开展投资环境推介、招商引资活动。创新招商方式，大力推进园区招商、产业招商、企业招商、以商招商和专业化、市场化招商，注重跨国并购、境外上市、服务外包等利用外资新方式。按照产业聚集、节能环保、财税增收等标准和要求，加快由招商引资到招商选资的转变，重点引进高新技术产业、先进制造业、现代服务业、节能环保、现代农业等领域的项目，引进世界500强企业、国有大企业、大型民营企业集团来我市设立生产制造基地、区域总部和技术研发中心。确保全年实际利用外商直接投资2.7亿美元以上。加强与韩国现代起亚汽车集团、新加坡裕廊集团、金鹰集团、中石化集团、中石油集团、中远集团、中海集团、中国建材集团、山东钢铁集团等国内外大企业的全面合作，积极推进与半岛城市群、鲁南经济带和大陆桥沿线城市的合作交流。抓住青岛海湾大桥、海底隧道建设的机遇，加快接轨青岛、一体化发展的步伐。

三是进一步优化投资环境。坚持围绕港口抓好基础设施建设，着力改善港口集疏运条件，提高港口利用效率和通过能力。年内完成港口建设投资24亿元，新建续建岚北港区一期航道、矿石码头二期、西区三期等10项港口建设重点工程。加快推进新菏兖日铁路电气化改造、日连盐铁路、黄岛至日照铁路、枣临岚铁路和岚山至临沂、日照至潍坊高速公路等工程，开工建设日照港连接同三高速公路的专用通道。加快电子口岸和鲁南（日照）国际贸易与航运服务中心建设步伐，争取设立国家级保税物流中心。建成220千伏后村等输变电工程。抓好重点园区规划建设，完善服务功能，增强承载能力。大力加强软环境和作风建设，深入推进公正执法、诚信服务，努力形成亲商、安商、扶商、富商的浓厚氛围。

四是加快转变外贸增长方式。加强政策引导，支持符合国家鼓励出口政策的企业发展。大力实施“科技兴贸”和“品牌带动”战略，重点发展机电产品和低能耗、低排放、高效益、高附加值的加工制品出口，培育出口品牌和优势企业。积极发展服务贸易，开展服务外包业务。抓住国家削减贸易顺差、调整进口关税等有利时机，扩大先进技术、重大装备、关键零部件及重要资源、原材料进口。积极发展对外经济技术合作，鼓励有条件的企业到国外投资办厂，开展工程承包、劳务合作，建立境外能源、资源基地。

（四）提升城市规划建设管理水平，建设生态宜居城市。更加注重城市的集约式发展，着力完善城市功能，突出生态特色，增加现代气息，加快建设有国际水准的滨海城市、全国领先的生态宜居城市和海内外知名的水上运动之都。

一是认真编制和严格控制城市规划。按照“双城一区、轴向拓展”的带状发展布局，完善城市规划体系，优化产业布局和功能区划，使港口、产业、园区、滨海特色相得益彰，设施、格调、景观、城市面貌互为辉映。以城市总体规划为依据，高起点编制各类专业规划、分区规划和详细规划，实现城乡建设规划全覆盖。年内重点编制好抗震防灾、防洪排涝、市级文化中心、城市色彩、户外广告等专业和专项规划。对不符合城市规划和生态要求的，坚决依法清理整治，切实维护规划的权威性和严肃性。

二是提高城市建设管理水平。按照“充实新市区、改造老城区、突破结合部”的思路，加快城市建设由规模膨胀向内涵提高转变。推进集中建设、联片发展，提高集约化建设水平。搞好城市总体、分区、项目的空间设计、环境设计、形象设计，提高建设品质，逐步推进标志性建筑物和标志性建筑群建设，充分体现滨海城市新风貌。加强城市综合执法和文明执法，实施全方位、全天候、网格化、数字化管理，健全城市管理长效机制，推进城市管理现代化。

三是加强城市基础设施建设。多方筹资43亿元，实施6项城市建设重点工程、9项城乡环境综合整治项目和4项市政公用事业配套工程。其中新建和改造山东路、淄博路等45条、91.1公里市区道路，进一步优化城市交通网络；完成市第一污水处理厂再生水利用工程、第二污水处理厂配套主干管网、经济开发区污水处理厂及市垃圾处理厂二期工程建设，构筑完善的城市治污体系；开工建设胶州至日照天然气长输管线工程，搞好供电、通信、燃气、供水、供热配套管网建设，进一步增强城市载体服务功能。

四是全面提升生态人居环境质量。深入开展城乡环境综合治理，继续加大对老城区、城乡结合部、背街小巷、居民社区整治力度。积极推进“城中村”改造，年内启动20个村居的改造工程。投资800万元，支持新农村试点示范村镇规划建设。抓好滨海地带、沿海公路、河道、山体、道路绿化工程，年内新增城区绿化面积300万平方米，造林4万亩。以贯彻市政府三个通告为契机，严格落实对山体、绿地、滨海湿地、河流、森林、岸线及海域等生态环境资源的保护措施。加强海岸带综合整治，严肃查处违法建设、破坏防护林和湿地、盗采海砂、违法排污行为，有计划地实施海岸带生态环境整治保护工程。加强对日照水库等城市饮用水源地保护，深入整治石材加工污染和农业面源污染，加大沭河、傅疃河、绣针河、洪凝河、崮河等流域综合治理力度，确保主要河流水质达到水功能区要求。积极争创“联合国人居环境奖”。

各位代表，“蓝天、碧海、金沙滩”是日照引以自豪的生态资源，也是广大人民共同的福祉。我们一定要以对人民、对子孙后代、对历史高度负责的态度，坚定不移地坚持环保优先、推进生态建市，让全市人民享有更广的蓝天碧海、更多的青山净水、更好的生态环境！

（五）努力改善民生，开创和谐社会建设新局面。坚持以人为本，从解决人民群众最关心、最直接、最现实的利益问题入手，着力保障和改善民生，努力让全市人民学有所教、劳有所得、病有所医、老有所养、住有所居、低有所保、困有所救。

一是加强就业和社会保障工作。全面落实积极的就业政策，加强政府引导，完善市场就业机制，促进以创业带动就业。实施就业困难人员援助工程，努力实现城乡“双零就业”家庭“动态消零”。全年新增城镇就业3.85万人，帮扶各类就业困难人员实现就业1000人以上，失业人员再就业6000人，再就业率达到75%以上。认真贯彻《就业促进法》、《劳动合同法》，大力开发就业岗位，搞好就业培训，规范各类用人单位劳动用工制度，维护职工合法权益。扩大社会保险覆盖面，抓好非公有制经济组织人员、城镇灵活就业人员和农民工的参保工作。逐步做实个人账户，积极推进企业退休人员社会化管理服务。加快农村社会养老保险制度改革，扩大农民工工伤保险、被征地农民养老保险覆盖范围，保持城乡最低生活保障动态管理下的应保尽保。特别是根据物价上涨情况，及时按政策提高低保标准。完善最低工资保障制度，建立企业职工工资正常增长机制和支付保障机制，逐步提高企业退休职工待遇。

二是协调发展教育、卫生、文化等各项事业。坚持教育优先发展，统筹城乡教育和各级各类教育。投入1.6亿元，巩固免除城乡义务教育阶段学生杂费成果，对农村义务教育阶段学生免费提供教科书，落实好普通高校、高中段学校贫困家庭学生资助政策。进一步改善城乡办学条件，年内在市区新建1处、改造4处中小学，完成6万平方米的农村中小学危房改造任务。坚持公共医疗卫生的公益性，健全公共卫生服务体系，稳步推进医药卫生体制改革，着力解决群众“看病贵”问题。深入挖掘和丰富龙山文化、莒文化、海洋文化内涵，组织策划奥林匹克水上公园水上嘉年华文体活动，打造具有日照特色的文化品牌。加快公共文化服务体系建设，大力实施广播电视“村村通”、文化信息资源共享工程、乡镇村居文化阵地建设、农村公益电影放映、“农家书屋”和社区图书室建设工程。精心组织2008帆船热身赛、中日韩国际大帆船比赛和奥运圣火在我市的传递活动，做好第十一届全运会日照赛区筹备工作，积极争办第二届中国水上运动会。严格落实人口目标责任制，加强人口和计划生育工作，提高人口素质。深化社会公德、职业道德、家庭美德、个人品德教育，营造良好的文明环境。深入开展全民国防教育、双拥共建活动，加强民兵和预备役部队建设。进一步做好史志、妇女儿童、青少年和老龄工作，积极发展社会福利事业、慈善事业和残疾人事业，调动一切积极力量参与和谐社会建设。

三是全面加强社会管理。认真实施“五五”普法和依法治市规划，努力提高全体公民的法律素质和全社会法治化管理水平。深入推进“平安日照”建设，强化社会治安综合治理，建立网格化防控体系，加强城乡社区村居警务工作，依法防范和打击违法犯罪活动。加强和改进信访工作，妥善处理各类社会矛盾，最大限度增加和谐因素。坚持安全发展，严格落实安全生产责任制，坚决防止重特大安全事故发生。积极推进和谐社区建设，把城乡社区建设成为管理有序、服务完善、文明祥和的社会生活共同体。加强应急管理体系和平台建设，完善各项应急预案，提高应对各种公共突发事件的处置能力，努力为全市人民创造安全、稳定、和谐的社会环境。

四是努力解决群众切身利益问题。积极完善经济适用住房制度，加快经济适用房货币直补进度，将廉租住房补贴对象由低保户扩大到低收入住房困难家庭。加强房地产市场调控，增加中小套型、中低价位普通商品房的供应量，构建多层次、科学合理的住房供给体系，防止房价过快上涨。强化市场和物价监管，抓好粮、油、肉、菜等生活必需品的供给，确保市场不断档、不脱销。加强农产品、食品药品质量监管体系建设，保证人民群众饮食、用药安全。着力解决好医疗、教育、土地征用、房屋拆迁、企业改制、供气供热等方面存在的突出问题，坚决杜绝侵害群众利益的行为。要时刻把人民群众的安危冷暖放在心上，努力为群众办实事、办好事、解难事。对国家、省、市今年出台的各项民生政策，要坚决落实，决不拖延；对已为群众办的实事，要认真检查兑现，继续巩固提高；按照侧重农村农民、侧重困难群众、侧重基本公共服务的原则，今年要着力办好12项为民实事重点项目，让广大人民群众共享改革发展成果。

四、切实加强政府自身建设

各位代表！这次大会将选举产生新一届市政府领导班子，新一届政府肩负着新时期光荣而艰巨的历史使命和全市人民的重托，必须按照党的十七大提出的要求，牢固树立立党为公、执政为民的理念，坚持科学执政、民主执政、依法执政、廉洁执政，下大力气抓好政府自身建设，努力建设服务型政府。

（一）加强学习调研，增强服务能力。深入学习贯彻党的十七大精神，把思想统一到十七大精神上来，把力量凝聚到实现十七大确定的各项目标任务上来，自觉用十七大精神武装头脑、指导实践、推动工作。坚持深入基层和群众，及时调查解决关系发展全局的战略问题和广大人民群众最关心的利益问题，使政府各项决策和工作部署更加符合科学发展规律，更能体现广大群众意愿。坚持与时俱进，继续解放思想，着力转变不适应不符合科学发展的思想观念，坚决克服满足现状、不思进取的思想，因循守旧、固步自封的思想，惧怕困难、畏首畏尾的思想，在解放思想中真抓实干，在转变观念中破解难题，在更新思路中转变发展方式，不断提高科学发展、为民服务的本领。

（二）加快职能转变，强化公共服务。切实增强宗旨观念，把实现好、维护好、发展好人民群众的根本利益作为政府一切工作的出发点和落脚点。进一步理顺政府与企业、市场、社会的关系，在更好地履行经济调节、市场监管职能的同时，切实加强社会管理和公共服务职能，为人民群众提供更多、更优、更均衡的公共服务产品，加快实现基本公共服务均等化。完善公共财政体系，以发展社会事业和解决民生问题为重点，切实加大对“三农”、教育、科技、文化、就业和社会保障、医疗卫生、节能减排环保、公共安全、基层组织建设等公共服务、基础性领域的投入，把有限的财力用在为人民群众谋利益上，加快建设“民生财政”。

（三）推进管理创新，提高工作效能。围绕服务型政府建设，制定和落实政府为社会、为群众服务的各项工作规范。拓展行政服务中心的服务职能，强化充分授权，规范审批流程，完善服务方式，真正建成方便市民和企业的服务中心。深化行政审批制度改革，减少审批事项和收费项目，确保我市行政审批项目低于周边城市。深入贯彻落实《政府信息公开条例》，完善政务公开、办事公开、新闻发布等制度，让行政权力在阳光下运行。发挥好市长公开电话、市长信箱和信访工作的作用，进一步畅通民意渠道，落实为民服务责任制，及时让群众的合理诉求得到解决，合法权益得到维护。加强电子政务建设，完善政务信息网络平台，提高服务社会、服务公众的能力。

（四）坚持依法行政，促进规范服务。牢固树立依法行政理念，把尊重人民权利意识、职权法定和责权统一意识融入到政府工作的各方面。健全行政决策机制，完善重大事项集体决策、专家咨询、社会公示与听证、决策评估等制度，推进决策的民主化、科学化。认真落实《中华人民共和国各级人民代表大会常务委员会监督法》，坚持向人大及其常委会报告工作和向人民政协通报情况的制度，自觉接受人大及其常委会和人民政协的监督。认真做好人大代表议案、建议、批评、意见和政协委员提案的办理、落实工作，广泛听取民主党派、工商联、无党派人士和各人民团体的建议意见。加强审计监督、行政监察，重视支持新闻舆论监督和社会公众监督，着力增强政府的公信力。

（五）坚持廉洁从政，转变工作作风。认真贯彻党中央、国务院和省委、省政府关于反腐倡廉的各项规定，全面落实政府系统党风廉政建设责任制。在坚决惩治腐败的同时，更加注重治本，更加注重预防，更加注重制度建设，保证权力在有效的监督下运行。始终牢记“两个务必”，坚持求真务实，艰苦奋斗，勤俭办一切事业，着力建设节约型政府、节约型社会。认真对照八个方面良好风气的要求，全面加强政风建设，切实转变思想作风、领导作风和工作作风，力戒形式主义、官僚主义，反对弄虚作假、急功近利，以为民、务实、清廉的作风推动科学发展、促进社会和谐。

各位代表！日照的发展正处在继往开来的重要战略机遇期。面对新的目标、新的任务，我们深感使命光荣，责任重大。让我们紧密团结在以胡锦涛同志为总书记的党中央周围，在省委、省政府和市委的坚强领导下，高举中国特色社会主义伟大旗帜，解放思想，励精图治，团结一心，开拓进取，为早日跨入全省先进行列，实现富民强市新跨越，创造日照人民的幸福生活和美好未来而努力奋斗！
